# Novel Analytical Platform For Robust Identification of Cell Migration Inhibitors

**DOI:** 10.1038/s41598-020-57806-0

**Published:** 2020-01-22

**Authors:** Parinyachat Somchai, Kriengkrai Phongkitkarun, Patipark Kueanjinda, Supawan Jamnongsong, Kulthida Vaeteewoottacharn, Vor Luvira, Seiji Okada, Siwanon Jirawatnotai, Somponnat Sampattavanich

**Affiliations:** 1 Siriraj Laboratory for Systems Pharmacology, Department of Pharmacology, Faculty of Medicine Siriraj Hospital, Mahidol University, Bangkok, 10700 Thailand; 2 Department of Biomedical Engineering, Faculty of Engineering, Mahidol University, Nakhon Pathom, 73170 Thailand; 3 Department of Biochemistry, Faculty of Medicine, Khon Kaen University, Khon Kaen, 40002 Thailand; 4 Department of Surgery, Faculty of Medicine, Khon Kaen University, Khon Kaen, 40002 Thailand; 5 Division of Hematopoiesis, Joint Research Center for Human Retrovirus Infection and Graduate School of Medical Sciences, Kumamoto University, Kumamoto, 860-0811 Japan

**Keywords:** Wide-field fluorescence microscopy, Cell migration, High-throughput screening

## Abstract

Wound healing assay is a simple and cost-effective *in vitro* assay for assessing therapeutic impacts on cell migration. Its key limitation is the possible confoundment by other cellular phenotypes, causing misinterpretation of the experimental outcome. In this study, we attempted to address this problem by developing a simple analytical approach for scoring therapeutic influences on both cell migration and cell death, while normalizing the influence of cell growth using Mitomycin C pre-treatment. By carefully mapping the relationship between cell death and wound closure rate, contribution of cell death and cell migration on the observed wound closure delay can be quantitatively separated at all drug dosing. We showed that both intrinsic cell motility difference and extrinsic factors such as cell seeding density can significantly affect final interpretation of therapeutic impacts on cellular phenotypes. Such discrepancy can be rectified by using the actual wound closure time of each treatment condition for the calculation of phenotypic scores. Finally, we demonstrated a screen for strong pharmaceutical inhibitors of cell migration in cholangiocarcinoma cell lines. Our approach enables accurate scoring of both migrastatic and cytotoxic effects, and can be easily implemented for high-throughput drug screening.

## Introduction

By acquiring genomic lesions in the forms of mutations, chromosomal aberrations, or modification of epigenetic marks, transformed (cancer) cells gain abilities to replicate, and eventually invade through the surrounding tissue boundaries. While cancer cells may acquire different phenotypic hallmarks^1^, ability of cancer cells to migrate out of their original niche is the major cause of cancer aggressiveness that underlies the demise of most cancer patients.

In recent years, pharmaceutical industries have gained more attentions in developing drug candidates that can specifically target molecular mechanisms related to cell motility, cell migration and metastasis^2^. Such ‘antimigrastics’, or drugs with ability to prevent cancer invasion and metastasis, can be complementary to the existing cytotoxic drugs that only focus in inducing tumor shrinkage. To identify candidate compounds that target cancer cell invasion, drug screeners often require *in vitro* assays for scoring therapeutic influence on cell migration. Wound healing assay is one of the most commonly used bioassays for evaluating the therapeutic impact on cell migration mainly due to its simplicity in experimental setup and data analysis at post processing. By scratching cell monolayer to create a wound, one can consistently perform wound healing assays across thousands of treatment conditions. Automated imaging platform with environmental control recently makes possible real-time monitoring of the wound closure, allowing quantification of wound closure rates to be easily assessed. One major challenge in quantifying the therapeutic impacts on cell migration is the interdependent roles of different cellular phenotypes during wound closure^3^. Prior studies have tried to suppress influence of cell growth on wound closure by pre-treating cells with Mitomycin C (MMC) or growing cells in low serum condition prior to drug treatment^4–6^. Cytotoxicity associated with most anti-cancer drugs is another confounding phenotype, which can cause an apparent delay in wound closure^2,7^. Because of such complexity, some drug candidates were mistakenly interpreted as cell migration inhibitors, although they were later found to be cytotoxic drugs in different cell lines or other treatment conditions^2^. An experimental approach that can separate the pharmacological impacts of different phenotypes will help ensure accuracy in screening for cell migration inhibitors while maintaining the simplicity of the conventional wound healing assay.

To address this limitation, we present in this study an analytical approach to improve the standard wound healing for accurate quantification of therapeutic impacts on different phenotypes. In addition to the ability to separate cell migration from cell death, our approach can robustly identify cell migration inhibitors across different cell lines at different seeding densities. We demonstrated the use of this new technique for identifying inhibitors of cell migration in cholangiocarcinoma cell lines. Our study offers a simple approach for quantitative scoring of both migrastatic and cytotoxic effects that can be readily scalable for high-throughput drug screening.

## Results

### Standard wound healing assay cannot accurately distinguish contributions of cell migration from cell growth or cell death

**Figure 1:**
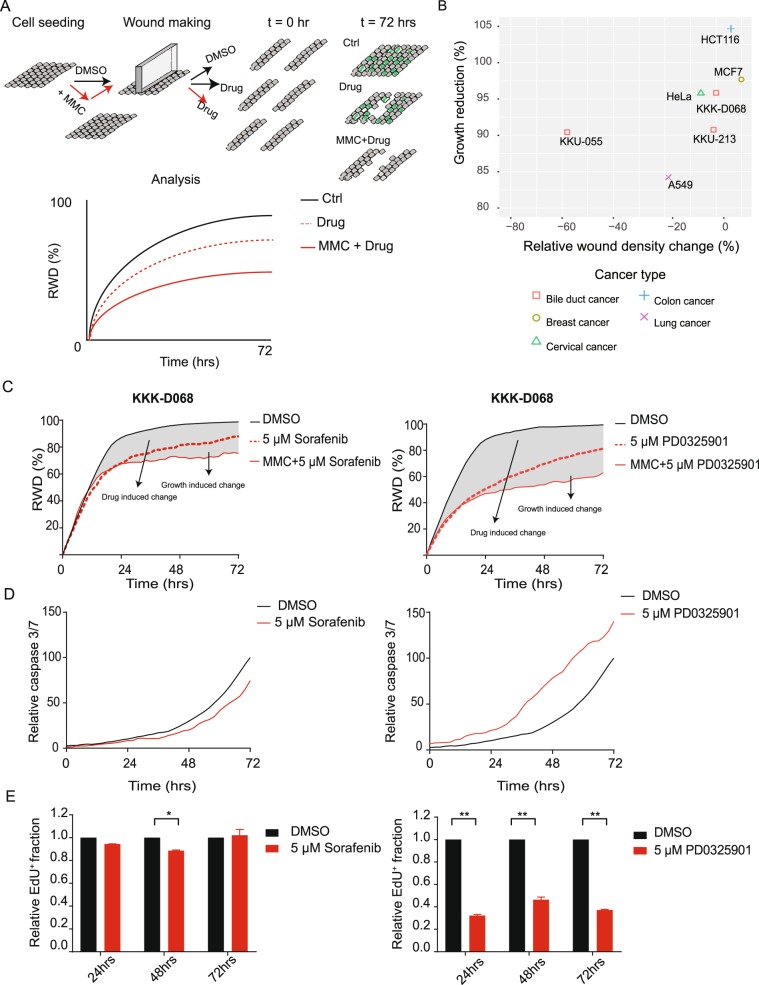
Complex confoundment of cellular phenotypes during standard wound healing assay. (**A**) Common procedures when implementing wound healing assay with or without Mitomycin C (MMC) pre-treatment. (**B**) Effects of Mitomycin C treatment on wound closure rate and cell proliferation across different cell lines. See also Supplementary Fig. S1A for example analysis of KKU-055, KKK-D068, and HCT116. (**C**) Measurement of RWD changes of KKK-D068 cell line treated with either Sorafenib (5 µM) or PD0325901 (5 µM), with (red solid line) or without (red dot line) MMC pre-treatment, in comparison with DMSO control (black solid line). (**D**) Relative caspase 3/7 intensity of KKK-D068 treated with Sorafenib (5 µM) or PD0325901 (5 µM) (red) in comparison with DMSO control (black). AUC was used for comparison between different conditions. (**E**) Relative fraction of EdU^+^ cells from similar conditions at 24, 48, and 72 hours, All data points were averaged from 2 biological replicates, (* vs **) represent significant differences of relative EdU^+^ fraction (p < 0.05 and 0.01, respectively).

The standard wound healing assay is one of the commonly used assays for assessing therapeutic impacts on cell migration although it is known to be confounded by complex interdependent roles of different cellular phenotypes^5,8^. To minimize impact of cell growth when adopting the standard wound healing assay, one often pre-incubate cells with MMC (3–5 hours) to limit cell growth, prior to assessing drug involvement on wound closure (Fig. 1A). We preliminarily assessed how effective the MMC pre-treatment protocol could inhibit cell proliferation and whether it also affects the rate of wound closure in different cell lines. We found that MMC pre-treatment protocol significantly affect the wound closure rate in some cell lines such as KKU-055 (cholangiocarcinoma) and A549 (lung) in addition to prohibiting cell proliferation (Fig. 1B). This result is not ideal since it implies that MMC pre-treatment itself can perturb cell migration in some biological models. Choosing appropriate cell lines for drug screening is thus a critical step when we implement MMC pre-treatment protocol, to ensure minimal influence of cell growth and accurate scoring of therapeutic impact on cell migration.

We next attempted to compare therapeutic impacts of two distinct compounds, sorafenib (pan-receptor tyrosine kinase inhibitor) and PD0325901 (potent MEK1/2 inhibitor) on cellular phenotypes during the use of wound healing assay. The KKK-D068 bile duct cancer cell line was chosen for this experiment because MMC pre-treatment could successfully inhibit its proliferation without affecting the baseline wound closure rate (Fig. 1B). At 5 μM which is a typical drug concentration for screening phenotypic effects, both compounds showed delays of wound closure, although PD0325901 exhibited more pronounced changes (the change of area under the curve [AUC] due to PD0325901 treatment is > 1.5 fold higher than that of Sorafenib) (Fig. 1C). To determine the cellular phenotypes that underlie the observed delay in wound closure, we quantified, using separate assays, impacts of each drug on apoptosis with caspase 3/7 activity kit and on cell proliferation with the EdU incorporation assay (Fig. 1D, E). Sorafenib showed no significant changes of caspase 3/7 activity (p = 0.22), while PD0325901 caused a significant increase in cell apoptosis (p = 0.0017) (Fig. 1D). EdU incorporation assay exhibited reduction of proliferative cells after PD0325901 treatment (p = 0.0047, 0.0018 and 0.00027 at 24, 48, and 72 hours, respectively), whereas Sorafenib showed negligible effects on EdU incorporation (p = 0.198, 0.024, and 0.477 at 24, 48 and 72 hours, respectively) (Fig. 1E). These results implied that the changes of wound closure rate from PD0325901 treatment must be contributed significantly by cell death. On the other hand, Sorafenib did not cause significant changes of cell proliferation or cell death; therefore, it could be inferred as a potential inhibitor of cell migration. These experiments demonstrate the typical inference of therapeutic effects on cell migration using the standard wound healing assay. And even with the implementation of MMC pre-treatment protocol, the quantitative therapeutic impact on cell migration still could not be quantitatively measured. An analytical framework that can quantify the confounders involved in the standard wound healing assay will enable accurate scoring of cell migration during primary drug screening.

### Concurrent measurement of cell death and wound closure rates enables quantitative scoring of different phenotypic contributions

**Figure 2:**
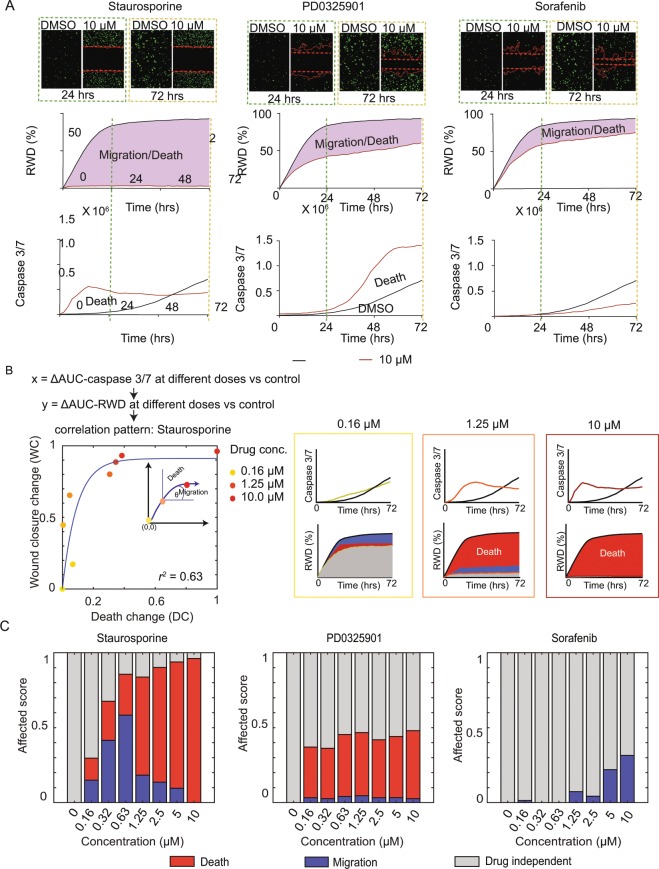
Relationships between changes of cell death and wound closure rates at different drug dosing. (**A**) Concurrent measurement of wound closure rates and caspase 3/7 signal from KKK-D068 cells treated with Staurosporine, PD0325901, and Sorafenib in comparison with DMSO control. Examples images at 24 and 72 hours (top) are shown together with the calculated relative wound density (middle) and caspase 3/7 signal (bottom). (**B**) Relationship of ‘wound closure change’ and ‘cell death change’ at different concentrations of Staurosporine (left), shown with examples of migration and death scores at three increasing drug concentrations (right). (**C**) Comparison of cell migration and cell death scores when treating KKK-D068 with Staurosporine (left), PD0320591 (middle) and Sorafenib (right).

We next attempted to understand how different cellular phenotypes contribute towards the observed delay of wound closure when we implemented wound healing assay for drug screening. We took advantage of the automated time-lapse microscopy to monitor cell death directly from our experimental setup of wound healing assay using the caspase 3/7 dye for apoptosis measurement (Supplementary Fig. S1B). To ensure no contribution of cell proliferation, we still applied the MMC pre-treatment protocol at the beginning of the experiment and selected the KKK-D068 cell line as our experimental model. We monitored changes of wound closure together with apoptosis caused by three drugs: Staurosporine, PD0325901, and Sorafenib, each at 8 concentrations ranging from 0.1–10 μM (Fig. 2A). At the highest drug concentration (10 μM), Staurosporine and PD0325901 showed significant cell death which could be observed concurrently with the delay of wound closure (Fig. 2A, left and middle panels). For Sorafenib, we again observed no effect on cell death although wound closure rate was slightly reduced (Fig. 2A, right panel). Since cell proliferation is suppressed by MMC pre-treatment in KKK-D068 cells, we assumed that the observed delay of wound closure in this cell line can be caused by either cell death or cell migration. By plotting the relationship between the cumulative changes in AUC of caspase signal and those from the relative wound density (RWD) changes at different drug concentrations, we were able to generate the dose-dependent multi-phenotypic contributions of each drug (Fig. 2B; also see Supplementary Fig. S2A for similar results from PD0325901 and Sorafenib treatment). With Staurosporine treatment, we observed a gradual increase of both cell death and wound closure changes at small drug doses, inferring that cell death and cell migration both contributed towards the observed wound closure delay (Fig. 2B; left panel). Such changes gradually leveled off at higher drug concentrations. A similar outcome was observed with PD0325901 treatment (Supplementary Fig. S2A; left panel). Notably, the relationship between cell death and wound closure is distinctly different in Sorafenib treatment where we observed a straight vertical line in this apoptosis-wound closure landscape (Supplementary Fig. S2A, right panel). Based on this outcome, we reasoned that the vertical relationship should imply a sole phenotypic contribution by cell migration whereas an inclining relationship would imply partial influences by both phenotypes. To approximate the contribution of each phenotype, we tried to fit this dose-dependent relationship of wound closure and cell death using a simple 1^st^-order rate equation and determined the contributions of cell death or cell migration based on the angular contribution at any specific points along the fitted curve, namely the angle between the fitted curve towards the vertical axis for cell death and the angle between the fitted curve towards the horizontal axis for cell migration (Fig. 2B, small inset on left panel). With this approximation, we could assign scores of phenotypic contributions for both cell death and cell migration at different drug concentrations (Fig. 2B, compare the contributions of cell migration and cell death at three different concentrations of Staurosporine).

We applied the developed approach for comparison of therapeutic influences on different phenotypes by Staurosporine, PD0325901, and Sorafenib treatment on the KKK-D068 cell line (Fig. 2C). Although all drugs actively inhibited their respective kinase targets (Supplementary Fig. S3), we found that Staurosporine caused inhibition of cell migration up to approximately 1 μM and switched to promote cell death more dominantly at higher drug concentrations. PD0325901 mainly caused cell death at all drug concentrations. Sorafenib inhibited cell migration at higher drug doses. We compared the ability of our technique to capture the relationship between cell death and wound closure changes in more cell lines and drug treatment (6 drugs across 5 CCA cell lines) and found that our approach could give reasonable fitting quality across all conditions (with median R^2^ > 0.6) (Supplementary Fig. S2B).

### Normalizing the variability of wound closure time improves robustness of phenotypic scoring

**Figure 3:**
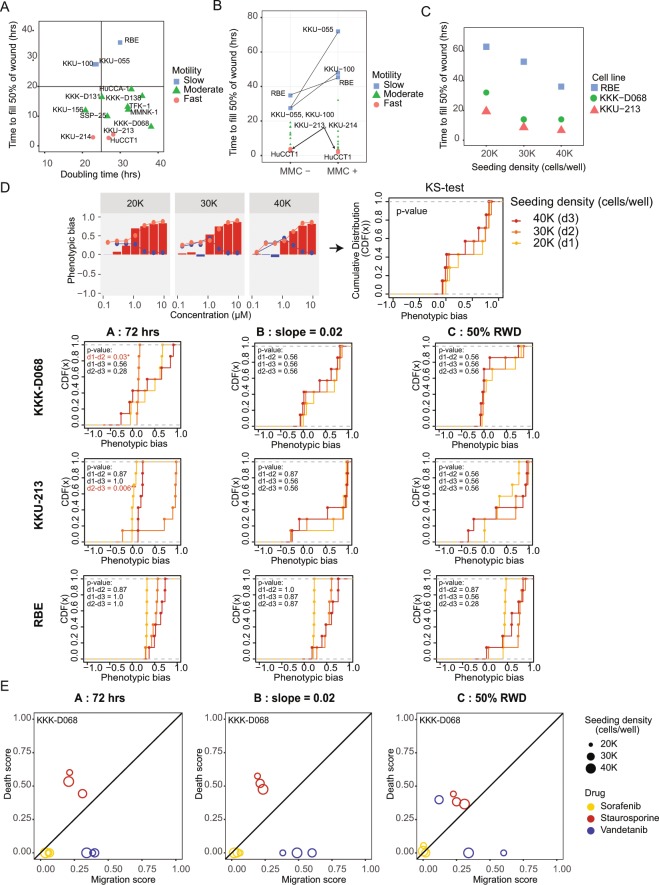
Contribution of intrinsic and extrinsic noises on the scoring of phenotypic contribution. (**A**) Intrinsic heterogeneity of cell motility across cell lines. Baseline wound closure rates (as measured by time to fill 50% of the generated wound) were compared against doubling times of CCA cell lines. (**B**) Comparison of wound closure rates (time to fill 50% of the wound) with or without MMC pre-treatment. (**C**) Influences of initial cell seeding density on wound closure rates. Wound closure time was compared among different cell lines with different initial seeding densities: 20 K, 30 K, and 40 K cells/well. (**D**) Comparison of measurement robustness using three different analytical endpoints. KS-test analysis was used to measure discrepancy of cumulative phenotypic biases among three different seeding densities (20 K, 30 K, and 40 K), from three different CCA cell lines. KS distances between different cell density pairs were measured and highlighted in red if the discrepancy is significant (p < 0.05). (**E**) Comparison of lumped phenotypic scores from three different endpoint criteria when KKK-D068 cells at different seeding densities (different marker sizes) were treated with Sorafenib (yellow), Staurosporine (red), or Vandetanib (blue).

Although the previous experiment gave us some evidence that the developed analytical approach works adequately to help score therapeutic influences on both cell migration and cell death, we wanted to ensure that the developed method can be applied to other biological models. When we compared the natural speed of wound closure time (i.e. no MMC pre-treatment) across 13 bile duct cancer cell lines and 1 cholangiocyte cell line (MMNK-1), we found that these cell lines could exhibit drastically different wound closure rates, ranging from 2.7 to 34.8 hours for closing 50% of the wound. Interestingly, the observed variability of wound closure rates was found to be independent of growth rate (quantified by doubling time) across cell lines (Fig. 3A). With MMC pre-treatment, some cells took longer time to close the wound (e.g. KKU-055, KKU-100, and RBE) as we previously showed, but the fast-moving cell lines including KKU-213, KKU-214 and HuCCT1 still take equally long for wound closure (Fig. 3B). In addition to the intrinsic variability of cell migration rate, we also explored how cell plating density, a potential extrinsic experimental artifact, may also influence wound closure speed. Choosing one cell line to represent each of the three classes of wound closure rate (KKU-213 for fast, KKK-D068 for intermediate, and RBE for cell line with slow wound closure rate), we compared the time that each cell line requires to close 50% of the wound when the cells were plated at different seeding densities, from 20,000 to 40,000 cells/well (Fig. 3C). In general, cells at higher seeding density filled the wound faster, although the intrinsic difference of cell motility across cell lines still dominated the impact of cell seeding density. Therefore, it is necessary that we ensure that the developed platform can give a robust interpretation of the therapeutic impacts on cellular phenotypes, regardless of these potentially variable intrinsic and extrinsic noises in different experimental setups.

To solve this issue, we investigated whether our approximation of migration and death scores can be made more robustly by choosing a more appropriate analytical period for each treatment condition. In particular, we evaluated 3 different endpoint criteria for our analysis of phenotypic scores: A) using the experiment endpoint (72 hours) as previously done, B) using the time that the rate of wound closure is declining (slope of the RWD < =0.02 for the no-drug control) and C) using the time where 50% of the wound is filled for each condition. For comparison, we monitored changes of wound closure speed using three different cell lines (KKK-D068, KKU-213, and RBE), each plated at 20,000, 30,000, and 40,000 cells/well (Supplementary Fig. S4A). Choosing Staurosporine as our test compound, we then determined cell death and cell migration scores at each drug concentration using these different endpoint criteria (Supplementary Fig. S4A; blue and red solid lines represent cell migration and cell death scores at different drug concentrations, respectively). Phenotypic bias (death score – migration score) was calculated at each drug concentration for comparison of robust scoring among the three different analytical approaches (Supplementary Fig. S4A; blue bars represent migration bias whereas red bars show cell death bias). Specifically, we used the Kolmogorov–Smirnov test (KS-test) to measure the distance of the cumulative phenotypic bias across all cell densities (Fig. 3D and Supplementary Fig. S4B). Method A showed the largest discrepancy of phenotypic bias among the different cell seeding densities while method B and method C were more agnostic to cell density changes (*p-value* > 0.05) consistently across all cell lines (Fig. 3D). We then tested how well the three endpoint criteria can be used to approximate phenotypic scores for other drugs, namely Sorafenib and Vandetanib. Instead of comparing phenotypic bias, we lumped the cell migration and cell death scores across concentrations into single cumulative scores, by calculating the normalized AUC for each phenotype score (Supplementary Fig. S4C). For the KKK-D068 cell line, the lumped migration and death scores from method B showed the most robust inference of therapeutic impacts across all seeding densities and all three drugs, while the scores showed scattered and non-uniform distributions when we used method A or C (Fig. 3E; see similar results for KKU-213 and RBE in Supplementary Fig. S4D). These results showed that using method B as our analytical endpoint can help minimize extrinsic artifacts from cell seeding density, ensuring that our approach can give more robust inference of different phenotypic contributions.

### Concurrent screening of both migrastatic and cytotoxic drug candidates for cholangiocarcinoma

**Figure 4:**
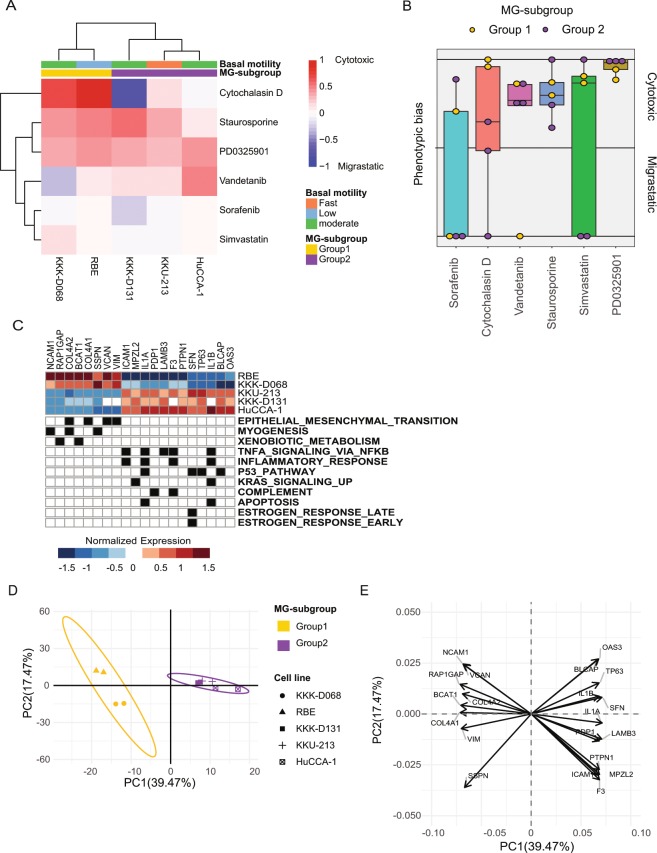
Compound screening for migrastatic and cytotoxic effects in cholangiocarcinoma cell lines. (**A**) Comparison of the phenotypic bias magnitudes. Blue and red grids represent compounds with migrastatic and cytotoxic effects, respectively. Cell lines are also annotated with their baseline motility (fast in red, moderate in green, and slow in blue), as well as their subgrouping based on hierarchical clustering (MG-subgroup 1 in yellow and MG-subgroup 2 in purple). (**B**) Comparison of the angular phenotypic bias across all six compounds. Different data points represent data from different CCA cell lines, also annotated here based on their MG-subgroups. (**C**) Development of subgroup classification biomarkers. Twenty leading-edge genes across different cancer hallmarks were identified using GSEA analysis for separating MG-subgroup 1 and MG-subgroup 2 cell lines. (**D**) PCA analysis of gene expression showing similarity among the 5 different CCA cell lines with ovals showing 95% confidence boundaries. (**E**) Loading plot showing eigenvectors of top-20 leading edge genes.

We next attempted to apply the developed platform for identifying drug candidates with either migrastatic or cytotoxic effect in cholangiocarcinoma cell lines. Using method B (declining slope) to determine the migration and death scores, we compared the phenotypic bias of six previously developed anti-cancer compounds across five CCA cell lines (HuCCA-1, KKK-D068, KKK-D131, KKU-213, and RBE). We reasoned that different drugs may cause similar phenotypic bias but the strength of the therapeutic impact could potentially be variable (compare A vs. D or B vs. E in Supplementary Fig. S5A). To compare the similarity of phenotypic bias for different drug candidates, we, therefore, developed two measures: 1) based on the actual distance away from the no-bias line and 2) based only on the angle of each condition away from the no-bias line (Supplementary Fig. S5B). First, when comparing the magnitude of phenotypic bias, we found that each drug can exhibit different phenotypic outcomes across different cell lines (Fig. 4A). For example, Cytochalasin D caused migrastatic lumped response in KKK-D131 and HuCCA-1 but gave cytotoxic lumped response in KKK-D068, RBE, and KKU-213. More interestingly, different drugs can inhibit both cell migration and cell death at different concentrations. For example, in KKK-D068, Staurosporine exhibited migrastatic effect at lower drug concentrations, but caused cytotoxic response at higher doses; a similar effect was observed in HuCCA-1 with Vandetanib treatment (Supplementary Fig. S5C). We next compared the phenotypic bias across the different drug candidates without considering the magnitude of the phenotypic bias. Specifically, the angle of phenotypic bias was used instead of measuring the actual distance from the no-bias line. PD0325901 and Staurosporine exhibited a uniform cytotoxic effect while Sorafenib showed migrastatic response from three out of five cell lines. Similarly, Simvastatin showed migrastatic response from two out of five cell lines with mild magnitude of the response (Fig. 4B, Supplementary Fig. S5C). Vandetanib caused migrastatic response only in KKK-D068. Cytochalasin D exhibited a cytotoxic response in most cells except KKK-D131 and HuCCA-1 that show migrastatic response.

Based on the similarity of phenotypic bias, we could group these cell lines into two major subgroups: (1) KKK-D068 and RBE, and (2) HuCCA-1, KKU-213, and KKK-D131 (Fig. 4A). We were interested to examine potential similarity of underlying molecular wiring among cell lines within the same phenotypic subgroup, based on basal transcriptomics. Using previously established gene sets of cancer hallmarks^9^ as our reference, we identified 20 leading-edge genes from the significantly enriched hallmark gene sets (NES score > 1 with adjusted *p-value* and *q-value* < 0.05) that can be used to classify the two cell line subgroups. In particular, MG-subgroup 1 (RBE and KKK-D068) showed enrichment of genes associating with epithelial-to-mesenchymal transition (EMT), myogenesis and xenobiotic metabolism, while MG-subgroup 2 (HuCCA-1, KKU-213, and KKK-D131) showed enrichment of genes that are associated with estrogen response, apoptosis, p53, KRAS signaling, and inflammatory response (Fig. 4C). In addition to cancer hallmark gene set analysis, we performed GO term biological function analysis and the result showed that extracellular matrix organization term was enriched in MG-subgroup 1 cells while terms related to apoptosis were enriched in MG-subgroup 2 cells (Supplementary Table S2). Similarity in gene expression within each of the two subgroups can also be best illustrated using the principal component space (Fig. 4D). The expression of the previously identified 20 leading-edge genes can serve as biomarkers for classifying cancer cell lines with similar therapeutic response (Fig. 4E). These results demonstrate the capability of our platform for assessing the multi-phenotypic effects of different drug candidates, leveraging from our ability to quantitatively score phenotypic contributions from the standard wound healing assays. Together with transcriptomic profiling, our platform can be used for developing predictive biomarkers to accompany the identified drug candidates.

## Discussion

Wound healing assay has been used ubiquitously for the assessment of cell migration in biomedical research but was also known to be confounded by complex influences of multiple cellular phenotypes in wound closure. We have demonstrated in this study how to modify the standard wound healing assay for accurate scoring of different phenotypic contributions. We showed that the conventional wound healing assay cannot be used to quantify accurately the contributions of different phenotypes, often leading to misinterpretation of phenotypic influences by each compound. By combining the MMC pre-treatment protocol with concurrent monitoring of cell death, we showed that the therapeutic effects on cellular phenotypes can be quantitated based on the established relationship of wound closure rate and changes of cell death at different drug concentrations. Fractional contribution of cell death and cell migration can then be approximated from the slope of the fitted relationship. We demonstrated the use of this simple analytical approach for robust screening of both migrastatic and cytotoxic compounds in cholangiocarcinoma cell lines. In addition to identifying potential drug candidates for this rare cancer type, we demonstrated the heterogeneity of therapeutic influences across cell lines and identified common similarity of cell lines based on basal gene expression profiling. Our GSEA analysis of basal transcriptomics identifies cell apoptosis and cell migration as the major differential hallmarks between the two cell line subgroups, confirming the robustness of our platform in faithfully capturing therapeutic effects on different cellular phenotypes. We have made the developed tool available for public use at https://sisp.shinyapps.io/MGCalculator/.

The main advantage of our approach is its simplicity in scoring both of cell migration and cell death within a single assay. This is consistent with the recent focus of pharmaceutical industry to take into account polypharmacologic effects^10^, and current interest in drug repurposing^7,11,12^. Recent studies began to adopt the standard wound healing assay for screening multi-phenotypic responses of compounds with polypharmacologic effects^13^. Other studies tried to investigate the effect of compounds or natural extract by integrating results from two independent phenotypic assays including both cell viability assay and wound healing assay^7,8,14,15^. However, such previous studies still required the primary screening to eliminate false positive compound with cytotoxic effect. The disadvantage in interpreting the therapeutic effects using separate assays is the inability to separate out compounds or natural extract that exhibit multi-phenotypic effects at different drug dosing. In contrast, our technique enable scoring of both migration and death impacts at different drug concentrations, and therefore can avoid misinterpretation of therapeutic effects due to drug toxicity as previously seen with Cytochalasin D or Latrunculin A^2^. In our own study to identify migrastatic drug candidates for CCA, Cytochalasin D exhibited heterogeneous phenotypic response in different CCA cell lines (Fig. 4). Therefore, the ability to score phenotypic effects as a function of drug dosing is critical for identifying truly migrastatic inhibitors that should exhibit increasing inhibitory effect on cell migration at elevated drug concentrations, instead of showing cytotoxicity.

Finally, most phenotypic assays are vulnerable to experimental artifacts such as cell plating density or intrinsic variability from different biological models. Measurement of drug response on cell viability has been re-visited recently. Different cell seeding densities have been shown to exhibit different growth rates, and such extrinsic variability can give rise to the conflicting conclusion of therapeutic response on cell viability from different laboratories. To rectify this problem, prior studies have come up with approaches to normalize the variable growth rates both from experimental artifacts like cell seeding density as well as the variable growth rate between cell lines^16,17^. For wound healing assay, we carefully characterized the influences of cell proliferation and cell seeding density on wound closure rate. While we observed that wound closure rate can be significantly affected by both intrinsic cell motility differences of different cell lines as well as extrinsic noise such as variability of cell seeding density (Fig. 3A–C), we showed that a simple modification of the analytical endpoint time can make our analysis more robust. Specifically, changing the assay endpoint time from the actual experimental endpoint (72 hours) to the time that the wound closure begins to saturate in each treatment condition can ensure more consistent inference of therapeutic effects (Fig. 3D, E).

One possible limitation of our technique is the inability to distinguish cell proliferation inhibitors from cytotoxic drugs since our analysis tried to normalize the contribution of cell proliferation by MMC pre-treatment. To rectify this problem, we perhaps can include scoring of growth inhibition in our platform by allowing cells to grow regularly and monitoring changes of cell count using nuclear staining or lineage tracer dyes, similar to the previous report^5,8^. An improved version of the platform could be set up without additional labelling steps, by using engineered cell lines with nuclear reporters together with caspase activity reporter. Such platform may not be as generalizable for all biological systems since it could be difficult to genetically engineer primary cell culture, or terminally differentiated cells. For generic drug screening platform, one can prepare engineered cell lines with different reporters for the most accurate scoring of different phenotypes, although the relationship between the contribution of each phenotype on wound closure will have to be reassessed individually.

## Methods

### Experimental methods and data processing

#### Cell line and culture condition

KKU-213, KKU-214, KKU-156, KKU-100, KKU-055, HuCCA-1, HuCCT1 and MMNK-1 were obtained from Japanese Collection of Research Bioresources (JCRB) Cell Bank, Japan. RBE, SSP-25, and TFK-1 were obtained from RIKEN Cell bank, Japan. KKK-D068, KKK-D131, KKK-D138 were obtained from Khon Kaen University, Thailand^18^. MCF7, HCT116, HeLa, A549 were purchased from ATCC. All cells were maintained at 37 °C in a humidified 5% CO_2_ atmosphere using growth media that were recommended by original cell sources (see complete details in Supplementary Table S1). The cell line panel consists of cancer of bile duct, which is known to be relatively migrative. Other cell lines with similar migratory property also include breast cancer (MCF7), lung cancer (A549), colorectal cancer (HCT116), and cervical cancer (HeLa). However, to test our novel method we selected a set of bile duct carcinoma cell lines known to be migrative as our model.

#### Drugs and dyes

Mitomycin C (S8146), Staurosporine (S1421), PD0325091 (S1036), Simvastatin (S1792), Sorafenib (S7397), Anisomycin (S7409), SP600125 (S1460) were purchased from Selleckchem. For Cytochalasin D (C8273) was purchased from Sigma-Aldrich and A443654 (HY-10425) was purchased from MedChemExpress. For drug treatment experiments, we treated cells at eight different concentrations using two-fold serial dilution starting from 10 µM. Each drug was dissolved first in DMSO. All treatment conditions contain the same final concentration of DMSO at 0.5% (v/v). Cell death was quantified using the CellEvent™ Caspase-3/7 Green dye (Thermo Fisher Scientific) at final concentration of 1% (v/v). For cell proliferation assay, cells were pulsed with ethynyl deoxyuridine (EdU) at the final concentration of 10 µM for 90 minutes. After cell fixation, EdU was tagged using click chemistry reaction containing 10 µM of carboxyrhodamine-110 azide dye, 4 mM of CuSO_4_ and 50 mM Ascorbic acid in 0.1 M Tris buffer at pH 8.5. Nuclear staining was performed using DAPI (Thermo Fisher Scientific).

#### Mitomycin C pre-treatment

Cells were seeded at specified densities and incubated overnight. Cells were then replaced with fresh media containing 5 µM MMC and incubated for 3 hours^19^. After being washed 3 times with 1xPBS, cells were incubated with specified conditions for 72 hours. At each time point (0, 24 and 72 hours), we then fixed the cells with 4% paraformaldehyde for 15 minutes at room temperature and stored in 1xPBS. For quantification of cell proliferation, cells were stained with DAPI (Thermo Fisher) and imaged with the Operetta high-content imaging system (PerkinElmer) at 10x magnification. Image analysis and object count were conducted via Columbus 4.8 (PerkinElmer).

#### Wound healing assay

Cells were seeded at specified densities on 96-well IncuCyte^®^ ImageLock Plates (Essen Bioscience) and incubated overnight. Growth media were replaced with fresh media containing 5 µM MMC and incubated for 3 hours. After being washed 3 times with 1xPBS and replenished with fresh media, cell monolayers were scratched to create a wound (700–800 micron wide) using the IncuCyte^®^ 96-well WoundMaker Tool (Essen Bioscience). Cells were then washed with 1xPBS three times and replaced with 100 µL fresh media. For concurrent monitoring of cell death, media were supplemented with CellEvent™ Caspase-3/7 Green dye (Thermo Fisher Scientific) at 1% (v/v). For drug screening, 10 µL of compounds were directly added to the cell medium to achieve specified final drug concentrations immediately after labeling with Caspase-3/7 Green dye.

#### Live microscopy

Cells with scratched wound were monitored every 2 hours for 3 days using IncuCyte imaging system^20^ (Essen Bioscience) at 10x magnification. To monitor wound closure rate, the percent RWD was generated at each time point for all treatment conditions. For measurement of cell death using the caspase 3/7 activity dye, integrated signal intensity was obtained from different treatment conditions using the same images acquired for the wound closure quantification.

#### Immunofluorescent staining and IC_50_ calculation

KKK-D068 cells were seeded at specified densities on CellCarrier-384 Ultra Microplates (PerkinElmer) and incubated at 37 °C, 5% CO_2_ overnight. Cells were then treated with different drugs or vehicle controls at specified conditions. For activation of ERK, AKT, and JNK signaling pathway, we used 100 ng/mL of EGF, IGF (AF-100-15 and AF-100-11, respectively, from PeproTech), and Anisomycin, respectively. For inhibition controls, we used 10 μM of PD0325901, A443654, and SP600125 to inhibit ERK, AKT, and JNK signaling pathways, respectively. Cells were treated with drugs for 6 hours followed by fixation using 4% paraformaldehyde for 15 minutes at room temperature and washed 3 times with 1xPBS. Then, the cells were permeabilized with absolute methanol for 1 hour at −20 °C followed by washing 3 times with 1xPBS. Prior to primary antibody staining, the cells were incubated with Odyssey® Blocking Buffer (LI-COR Biosciences™) for 1 hour at room temperature to block nonspecific protein binding. After that, anti-rabbit mAB specific to phospho-p44/42 MAPK (Erk1/2) (THR202/Tyr204) (1:300), phospho-Akt (Ser473) (1:300), or phospho-c-Jun (Ser73) (1:300) (#4370, #4060, #3270, respectively, Cell Signaling Technology) was added to the cells followed by an overnight incubation at 4 °C. On the next day, the cells were washed 3 times with 1x PBST followed by staining with donkey anti-rabbit IgG (H + L)-Alexa Fluor® 647 secondary antibody (1:1000) (#4414, Cell Signaling Technology) for 2 hours at room temperature. Afterward, the excessive secondary antibody was washed out 3 times with 1xPBST and once with 1xPBS prior to nuclear staining using DAPI (1:2000) (#D1306, Thermo Fisher) and whole-cell staining using HCS CellMask Green stain (1:1000) (#H32714, Life Science Technology) for 1 hour at room temperature. After 3 washes with 1xPBS, the cells were filled with 200 μl of 1xPBS and the plate was sealed. Cell images were collected at 10X magnification using CLS high-content imaging system (PerkinElmer) and segmented. The phosphorylation levels of kinases were reported as mean fluorescence intensity (MFI) using Columbus 4.8 software (PerkinElmer). For analysis of phosphorylation activity, MFI of kinases in nuclear compartment was used for p-p44/42 and p-c-Jun whereas MFI of kinases in membrane region was used for p-Akt. IC_50_ values that inhibit phosphorylation of kinases were calculated from drug concentration versus normalized MFI curve fit using GraphPad Prism version 8.0.1 for Windows (GraphPad Software).

### Metrics of multi-phenotypic responses

#### Calculation of cell migration/death scores

To separate the contribution of cell migration and cell death from the observed delayed in wound closure, we generated the relationship of the cumulative changes of cell death signal and the relative wound density.

For calculation of “wound closure change” (*WC*_*xi*_), we determined the AUC of the RWD from each drug treatment condition (*AUC(RWD)*_*xi*_) and normalized this value by that from the DMSO control group (*AUC(RWD)*_*ctrl*_), all with MMC pre-treatment, using the following formula.

$$W{C}_{xi}=(AUC{(RWD)}_{ctrl}-AUC{(RWD)}_{xi})/AUC{(RWD)}_{ctrl}$$

It is important to note that some drug cannot completely inhibit wound closure. For example, with PD0325901, the generated wound still close to 50% even at the maximum drug dose (Fig. 2A, middle panel). On the other hand, Staurosporine at 10 μM can inhibit wound closure almost entirely (Fig. 2A, left panel). To consider this difference, we define another parameter called drug-independent wound closure change (*WC*_*ind*_). We can summarize the relationship between the two types of wound closure by the following equation.

$$1=W{C}_{xi}+W{C}_{ind}$$

where

$$W{C}_{ind}=AUC{(RWD)}_{xi}/AUC{(RWD)}_{ctrl}$$

For “Death change” (*DC*_*xi*_), we determined the area under curve from caspase 3/7 activity (*AUC(D)*_*xi*_) at each drug treatment condition and normalized this value by that of the DMSO control (*AUC(D)*_*ctrl*_), both with MMC pre-treatment.

$$D{C}_{xi}=(AUC{(D)}_{xi}-AUC{(D)}_{ctrl})/AUC{(D)}_{max}$$

The data obtained from *WC*_*xi*_ and *DC*_*xi*_ were used to determine the contribution of cell death and cell migration from the drug-induced wound closure change. We attempted to capture this relationship by fitting with 1^st^-order rate equation. If the fitting quality is inadequate (i.e. *r*^2^ < 0.6), a straight line is used instead. If the fitting of straight line is favorable, we concluded that the observed wound change is dependent only on one cellular phenotype. An example of the linear fit is that from Sorafenib treatment where no cell death is observed at any drug concentration (Fig. 2A, right panel and Supplementary Fig. S2A).

When the relationship is explainable by 1^st^ order rate equation, we approximated the fractional contribution of cell migration and cell death by calculating the arctan of the slope (*θ*_*xi*_ in radian). The summation of the migration score (*Migration*_*xi*_) and death score (*Death*_*xi*_) should be equal to the drug-induced wound closure change (*WC*_*xi*_). Mathematically, such relationship can be defined as follows:

$${W}{{C}}_{{xi}}={Migratio}{{n}}_{{xi}}+{Deat}{{h}}_{{xi}},$$

where

$${Deat}{{h}}_{{xi}}={W}{{C}}_{{xi}}\times ({{\theta }}_{{xi}}/(\frac{{\pi }}{2})).$$

#### Phenotypic bias calculation

To determine the phenotypic bias, we can look at the landscape of *Migration*_*xi*_ (as x axis) and *Death*_*xi*_ (as y axis). In this landscape, the diagonal line (y = x) represents all conditions without any phenotypic bias, i.e. no-bias line. To measure the phenotypic bias, one can use the no-bias line as the reference, and calculate 1) the perpendicular distance away from the no-bias line or 2) the angular deviation away from the no-bias line. To calculate the perpendicular distance of each condition away from the no-bias line, we can prove mathematically that this distance is equal to $\sin (\frac{\pi }{4})$ (*Death*_*xi*_ − *Migration*_*xi*_) (Supplementary Fig. S5A). Therefore, we can use the normalized difference between death score and migration score to represents the magnitude of phenotypic bias. Alternatively, we can measure the angular deviation of the migration/death scores from the no-bias line, to measure the radial component of the phenotypic bias (Fig. 4B).

#### Transcriptome analysis, gene set enrichment analysis, and GO term analysis of MG-subgroup

Total RNA samples were extracted from CCA cell lines using Trizol-based protocol (Geneaid) and purified by RNA specific column (Invitrogen). The quality of RNA was evaluated by Nanodrop (Thermo Scientific), Qubit RNA BR kit (Invitrogen) and Bioanalyzer 2100 (Agilent Technologies). The RNA sequencing libraries were constructed following standard protocol using Illumina-compatible NEBNext® Ultra™ Directional RNA Library Prep Kit (New England BioLabs, MA, USA). The libraries were sequenced on an Illumina Hiseq2000 using paired-end reads (150-2 bp) at depth 20–25 million reads per sample. STAR method version v2.5.3a is performed to align RNA sequence^21^. Phred quality score (Q score) at 10 was used to filter data with low quality^22^. The measurement of gene expression, transcription per million (TPM), is used for only genes that have a median of TPM greater than zero. Gene expression data were log-transformed (log_2_ (TPM + 1)), and processed with R programming language^23^. Finally, RNA sequencing data were cleaned by surrogate variable analysis (SVA), before further analysis^24^. Prior to gene set enrichment analysis, average expression levels of genes in CCA cell lines identified as MG-subgroup 1 or 2 were calculated. We then converted the averaged expression level to fold-change level of gene expression of MG-subgroup 1 over 2, ranked the genes based on the fold-change levels from high to low, and performed GSEA using 50 cancer hallmark gene sets from MSigDB^9^ (version 6.2) and ‘clusterProfiler’ (version 3.10.1) R package^25^.

As a result, we obtained the cancer hallmark gene sets with normalized enrichment scores (NES) and p-values, representing ‘bird eye-view’ of the transcriptomic profiles of MG-subgroup 1 compared to subgroup 2. Significant cancer hallmark pathways were selected based on Benjamini-Hochberg adjusted p-value^26^ < 0.05 and FDR q-value^27^ < 0.05. After identification of significantly enriched gene sets, leading-edge genes, which highly contribute to NES, from each of the gene sets were collected. From this gene list, we identified 20 genes that were also expressed in our cell lines and further considered as potential MG subgroup biomarkers. Principle component analysis was also performed to confirm the correlation between the identified biomarkers and MG subgroups. These data were processed and visualized using ‘pheatmap’ (version 1.0.12) and ‘factoextra’ (version 1.0.5) R packages.

To analyze GO term biological functions of our novel MG subgroup biomarkers, the web-based enrichment tool Enrichr^28,29^ was used and we collected the enrichment results from ‘GO biological process 2018’ feature. Any GO terms with nominal p-value < 0.05 and FDR-adjusted p-value < 0.5 were excluded and we selected the top 5 GO terms from each MG subgroup.

## Supplementary information

Supplementary information

## Data Availability

Raw data from the current study are available at 10.6084/m9.figshare.8856974.
