# Supplementary material for: Novel Analytical Platform For Robust Identification of Cell Migration Inhibitors
Source: Sci Rep. 2020 Jan 22;10:931. doi: 10.1038/s41598-020-57806-0 (PMC6976598; doi:10.1038/s41598-020-57806-0)
Supplement: Supplementary file 1 — Supplementary information [file 41598_2020_57806_MOESM1_ESM.pdf]

Supplementary information

NOVEL ANALYTICAL PLATFORM FOR ROBUST IDENTIFICATION OF CELL MIGRATION INHIBITORS

Parinyachat Somchai[1], Kriengkrai Phongkitkarun[1,2], Patipark Kueanjinda[1], Supawan Jamnongsong[1], Kulthida Vaeteewoottacharn[3], Vor Luvira[4], Seiji Okada[5], Siwanon Jirawatnotai[1], Somponnat Sampattavanich[1,*]

[1] Siriraj Laboratory for System Pharmacology, Department of Pharmacology, Faculty of Medicine Siriraj Hospital, Mahidol University, Bangkok, Thailand
[2] Department of Biomedical Engineering, Faculty of Engineering, Mahidol University, Nakhon Pathom, Thailand
[3] Department of Biochemistry Faculty of Medicine, Khon Kaen University, Khon Kaen, Thailand
[4] Department of Surgery, Khon Kaen University, Khon Kaen, Thailand
[5] Division of Hematopoiesis, Joint Research Center for Retroviral Infection & Graduate School of Medical Sciences, Kumamoto University, Japan
[*] Corresponding author: Somponnat Sampattavanich, Tel. +6624195248 Email: somponnat.sam@mahidol.edu

Supplementary Figures

A

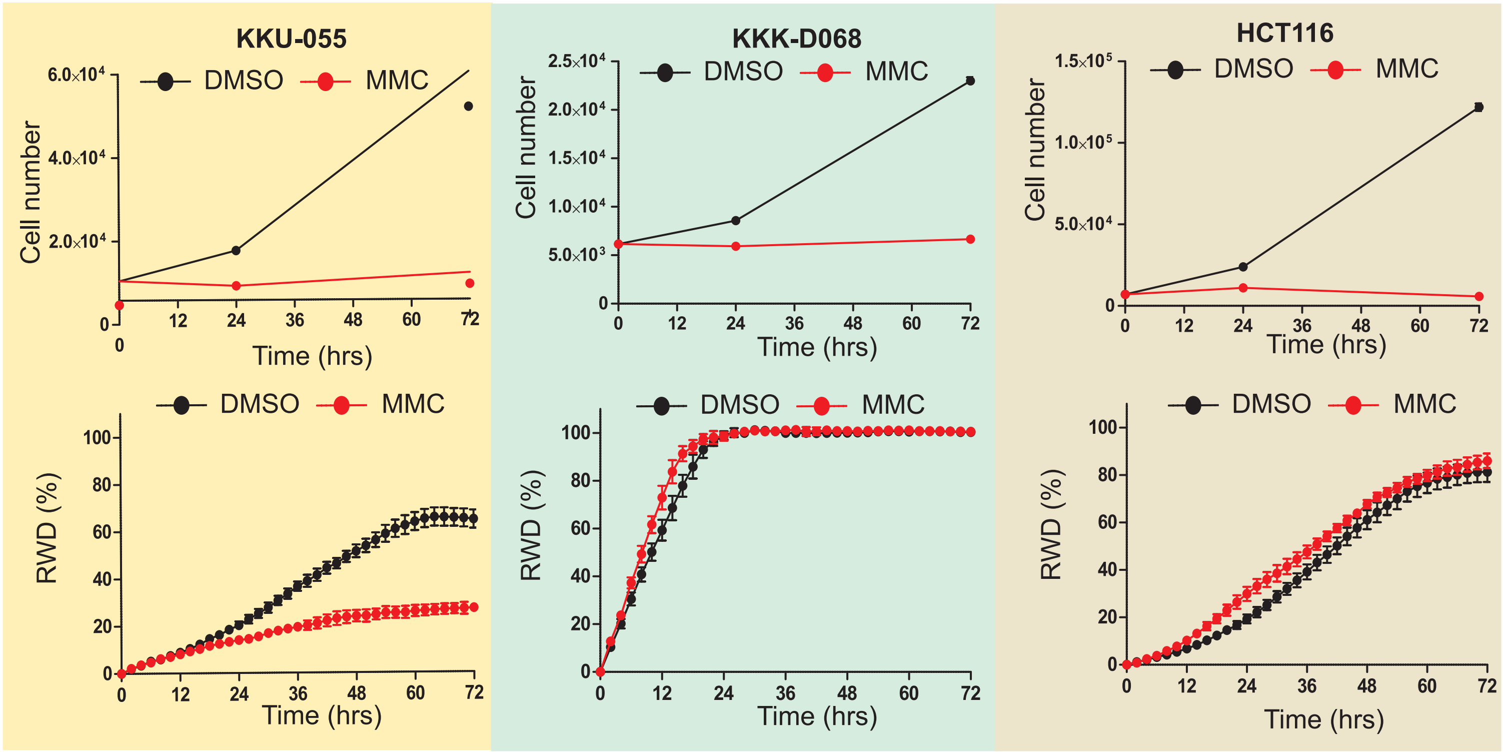

B

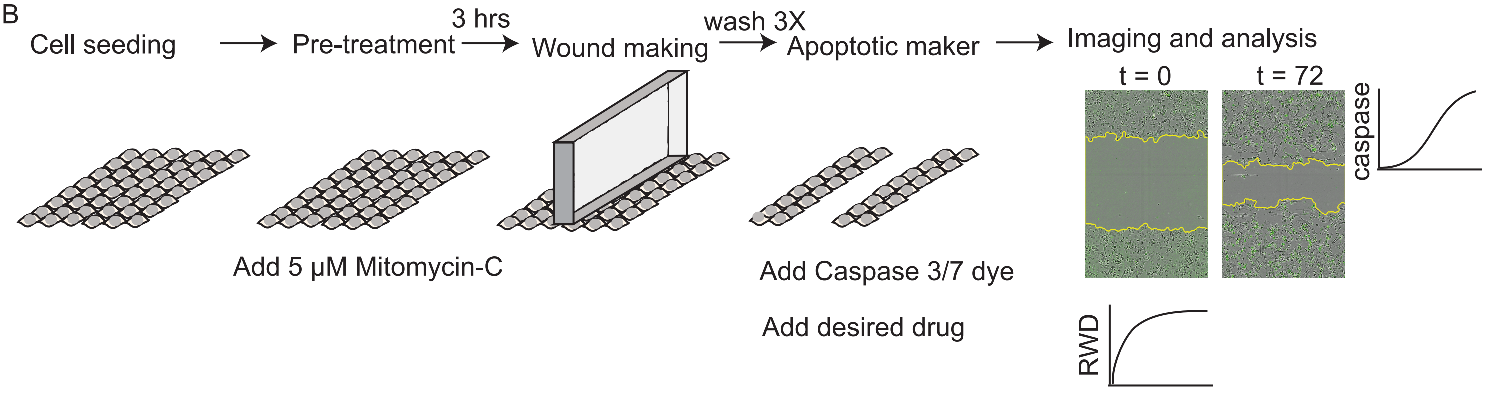

Figure S1. Characterization of wound healing assay. (A) Effects of Mitomycin C on wound closure rate and cell proliferation for KKU-055, KKK-D068 and HCT116. (B) Workflow for concurrent measurements of relative wound density and caspase 3/7 signal.

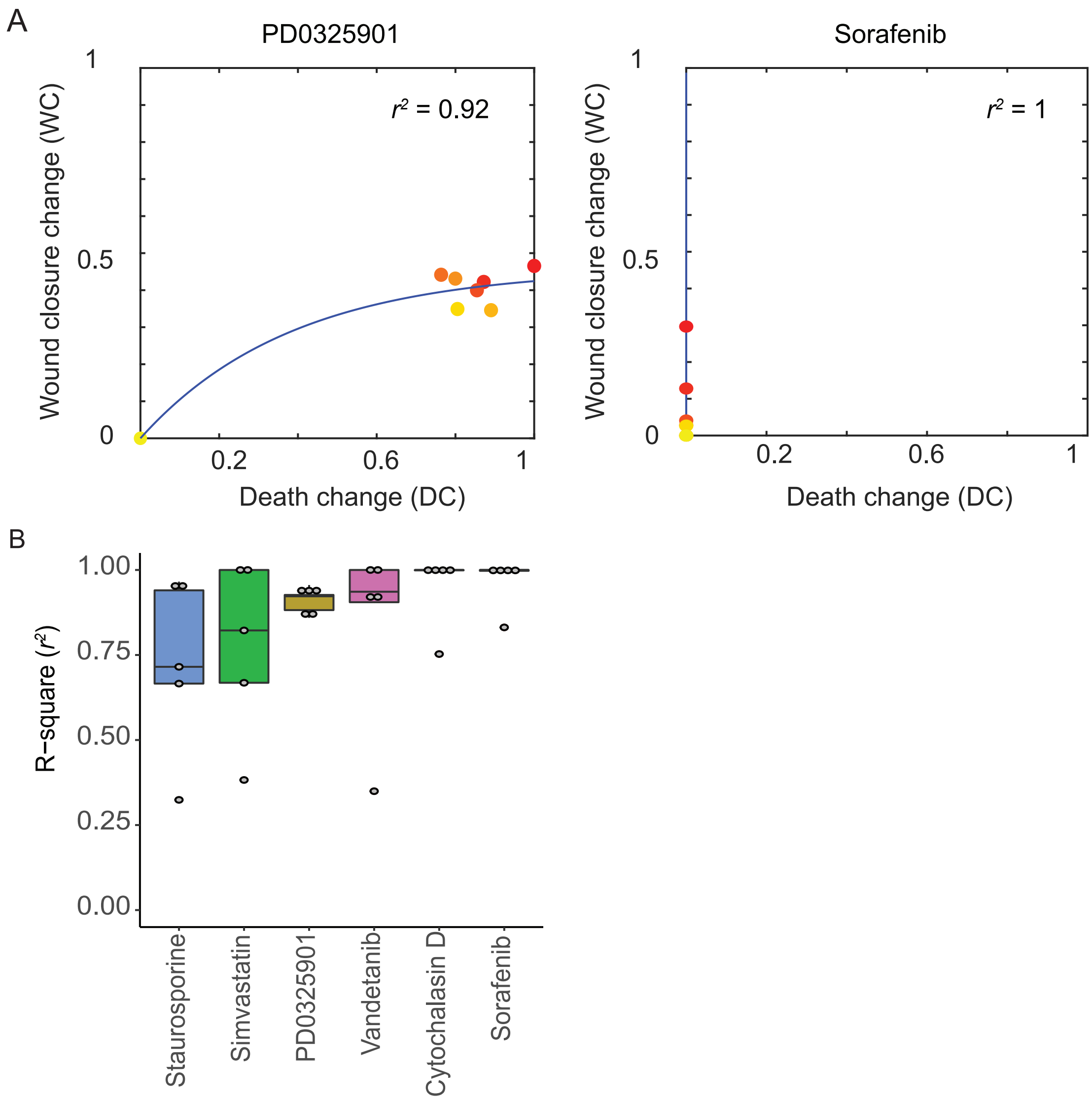

Figure S2. Evaluation of curve fitting quality. (A) Relationship of 'wound closure change' and 'cell death change' at different concentrations of PD0325901 and Sorafenib. (B) The quality of model fitting, as measured by the r^2, using the experimental endpoint: 72 hours for the analysis. Data were compared across 6 different compounds from five CCA cell lines (different dots).

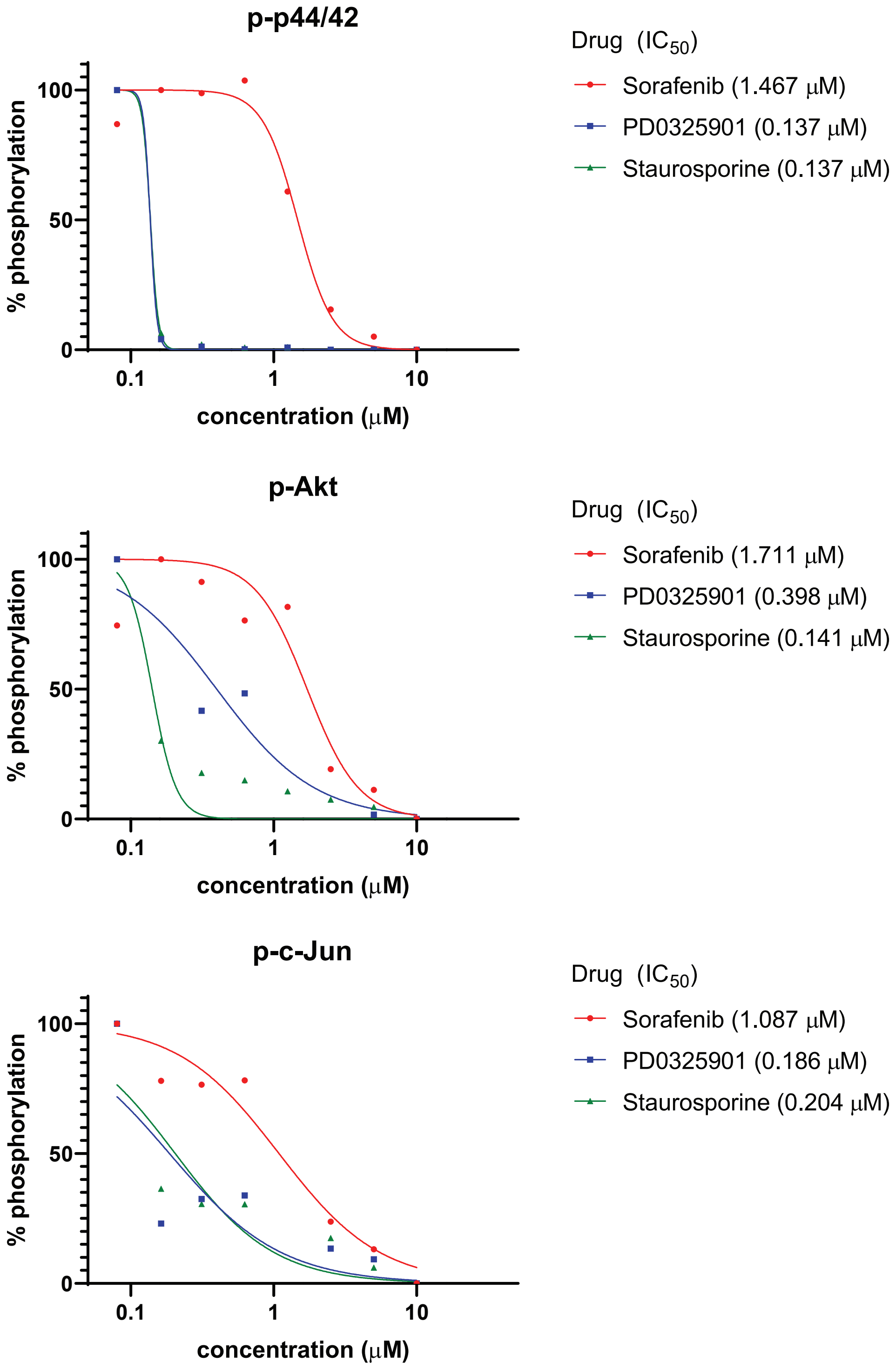

Figure S3. Dose-response changes of ERK, AKT, and JNK pathways in the KKK-D068 cell line at 6 hours after treatment with Sorafenib, PD0325901 and Staurosporine.

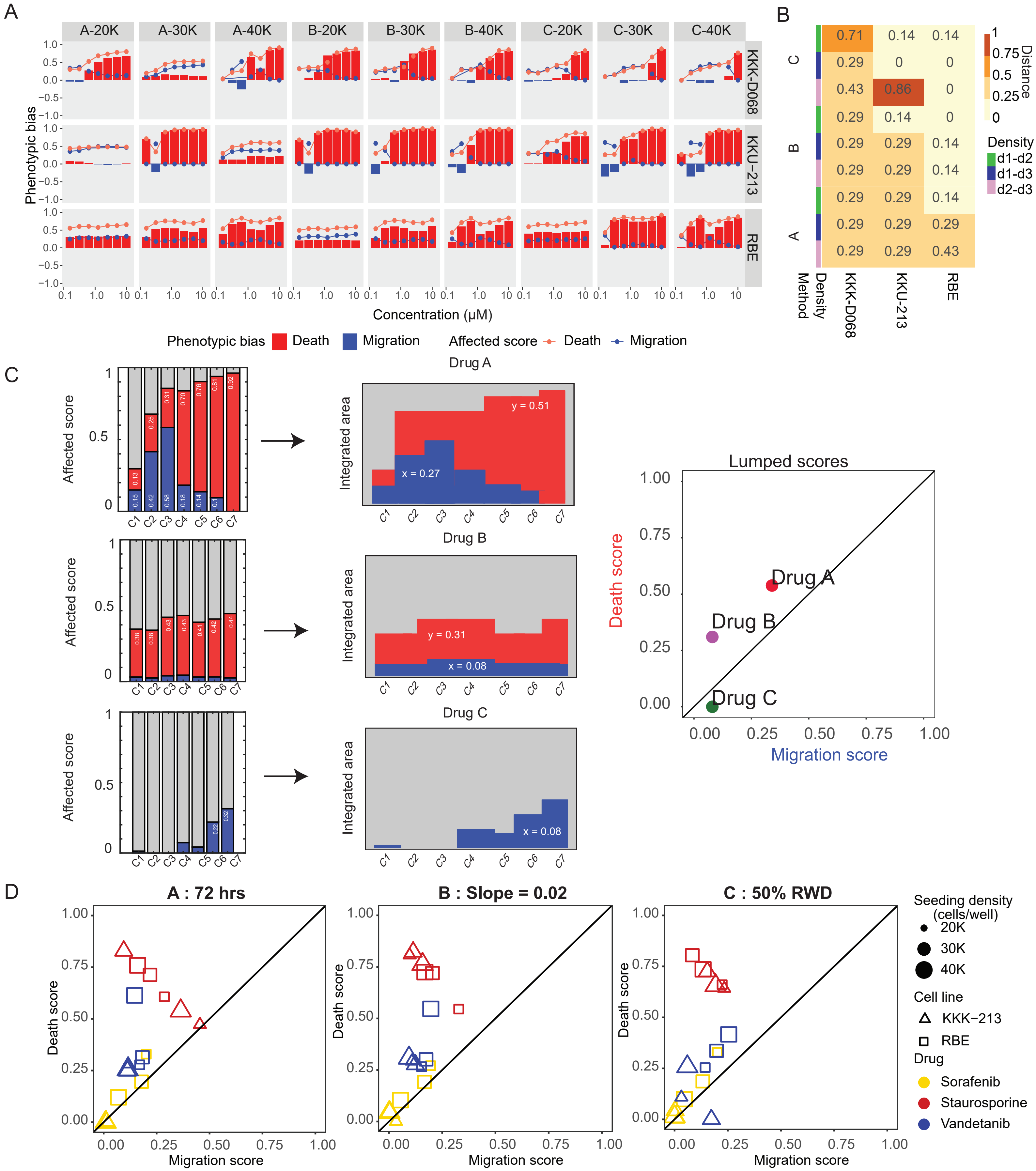

Figure S4. Identification of analytical approaches for robust scoring of phenotypic contributions. (A) Calculation of phenotypic bias of Staurosporine at different concentrations (0.1-10 μM) when different analytical endpoints (i.e. A) 72 hours), B) slope = 0.02), and C) RWD = 50%) were applied. (B) Comparison of the maximum KS distance for different pairs of seeding density (d1-d2, d1-d3 or d2-d3) from three cell lines generated using the three different endpoint criteria. (C) Diagram showing steps for calculating lumped scores for cell death and cell migration contribution for three example drugs. (D) Comparison of lumped migration or death scores when the three different endpoint criteria were used. Data were compared among three different compounds: Sorafenib (yellow), Staurosporine (red), and Vandetanib (blue), when cells (KKU-213 shown in triangles and RBE shown in squares) were plated at three different seeding densities (varying marker sizes).

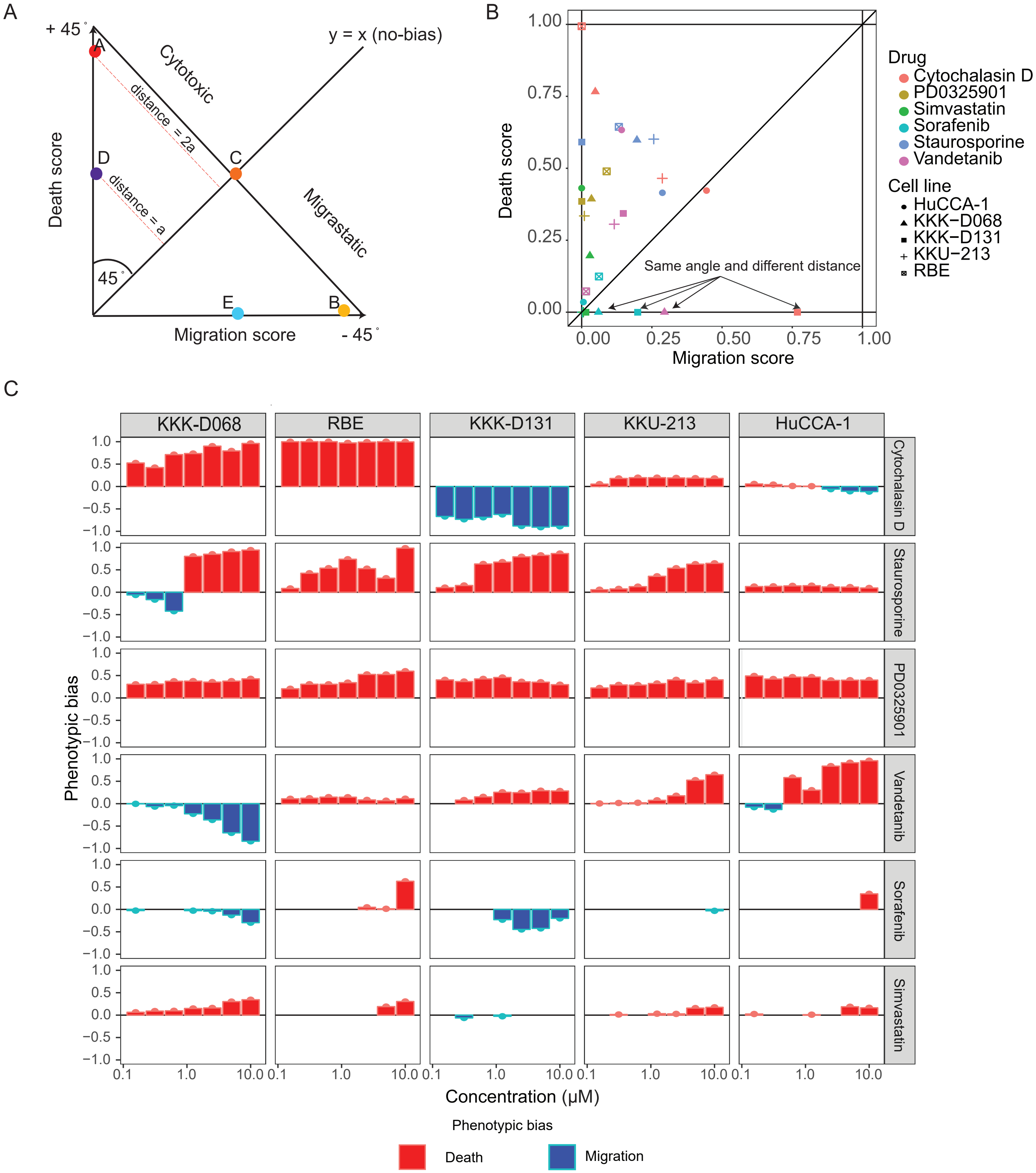

Figure S5. Phenotypic bias calculation. (A) Diagrams showing measurement of phenotypic bias magnitude and phenotypic bias angle from the no-bias line. Drug A and D exhibit distances of 2a and a respectively from the no-bias line, giving the same angular phenotypic bias of 45 degrees and therefore giving complete cytotoxic effect. Drug E and B show similar relationship, but with complete migrastatic effect. (B) Landscape of lumped migration and death scores for compound effects across all CCA cell lines. (C) Phenotypic bias as a function of drug concentration for different drug-cell line combination. See also Supplementary Table S3 for complete IC50 of both apoptosis and wound healing assay.

Table S1.

Cell culture and growth condition

Cell line	Growth media
KKU-156	DMEM supplemented with 10% FBS and 100 U/mL penicillin and streptomycin
KKU-213	DMEM supplemented with 10% FBS and 100 U/mL penicillin and streptomycin
KKU-214	DMEM supplemented with 10% FBS and 100 U/mL penicillin and streptomycin
KKU-055	DMEM supplemented with 10% FBS and 100 U/mL penicillin and streptomycin
KKU-100	HAMF12 supplemented with 10% FBS and 100 U/mL penicillin and streptomycin
HuCCA-1	HAMF12 supplemented with 10% FBS and 100 U/mL penicillin and streptomycin
HuCCT1	DMEM supplemented with 10% FBS and 100 U/mL penicillin and streptomycin
MMNK-1	HAMF12 supplemented with 10% FBS and 100 U/mL penicillin and streptomycin
RBE	RPMI1640 supplemented with 10% FBS and 100 U/mL penicillin and streptomycin
SSP-25	RPMI1640 supplemented with 10% FBS and 100 U/mL penicillin and streptomycin
TFK-1	RPMI1640 supplemented with 10% FBS and 100 U/mL penicillin and streptomycin
KKK-D068	DMEM supplemented with 10% FBS and 100 U/mL penicillin and streptomycin
KKK-D131	DMEM supplemented with 10% FBS and 100 U/mL penicillin and streptomycin
KKK-D138	DMEM supplemented with 10% FBS and 100 U/mL penicillin and streptomycin
MCF7	DMEM supplemented with 10% FBS and 100 U/mL penicillin and streptomycin
HCT116	DMEM supplemented with 10% FBS and 100 U/mL penicillin and streptomycin
HeLa	DMEM supplemented with 10% FBS and 100 U/mL penicillin and streptomycin
A549	DMEM supplemented with 10% FBS and 100 U/mL penicillin and streptomycin

Table S2.

GO term analysis of the selected 20 genes

	Description	FDR	Matching proteins
MG subgroup 1	collagen-activated tyrosine kinase receptor signaling pathway	0.003	COL4A1,COL4A2
	extracellular matrix organization	0.0236	COL4A1,COL4A2,VCAN
	nervous system development	0.0467	COL4A1,NCAM1,RAP1GAP,VCAN,VIM
	cell differentiation	0.0467	COL4A1,COL4A2,NCAM1,RAP1GAP,VCAN,VI
MG subgroup 2	regulation of apoptotic signaling pathway	6.60E-05	ICAM1,IL1A,IL1B,PTPN1,SFN,TP63
	acute inflammatory response	6.62E-05	F3,ICAM1,IL1A,IL1B
	fever generation	0.0014	IL1A,IL1B
	regulation of epidermal cell division	0.0014	SFN,TP63
	positive regulation of organelle organization	0.0014	ICAM1,IL1A,IL1B,SFN,TP63
	ectopic germ cell programmed cell death	0.0014	IL1A,IL1B
	regulation of apoptotic process	0.0014	F3,ICAM1,IL1A,IL1B,PTPN1,SFN,TP63
	negative regulation of apoptotic process	0.0014	ICAM1,IL1A,IL1B,PTPN1,SFN,TP63
	regulation of cell division	0.0014	IL1A,IL1B,SFN,TP63
	negative regulation of apoptotic signaling pathway	0.0014	ICAM1,IL1A,IL1B,PTPN1
	multicellular organismal homeostasis	0.0015	IL1A,IL1B,SFN,TP63

Table S3: IC50 of caspase activity and wound healing assay from various drugs and cell lines

Cell line	Drug name	End point (hr)	IC50 of caspase activity relative to no-drug ctrl	IC50 of relative wound density relative to no-drug ctrl
KKK-D068	staurosporine	24	0.9167	≤ 0.1625
KKK-D068	Sorafenib	24	≥ 10	1.676
KKK-D068	PD0325901	24	2.754	0.3079
KKK-D068	Simvastatin	24	≥ 10	≥ 10
KKK-D068	Vandetanib	24	≥ 10	9.652
KKK-D068	CytochalasinD	24	≥ 10	0.484
KKU-213	staurosporine	24	1.062	0.5236
KKU-213	Sorafenib	24	5.109	0.6126
KKU-213	PD0325901	24	≥ 10	≥ 10
KKU-213	Simvastatin	24	≤ 0.1625	2.39
KKU-213	Vandetanib	24	6.106	0.5112
KKU-213	CytochalasinD	24	0.6522	2.25
KKK-D131	staurosporine	24	3.585	≤ 0.1625
KKK-D131	Sorafenib	24	≥ 10	≤ 0.1626
KKK-D131	PD0325901	24	≤ 0.1625	0.8977
KKK-D131	Simvastatin	24	0.3124	≥ 10
KKK-D131	Vandetanib	24	≥ 10	≤ 0.1625
KKK-D131	CytochalasinD	24	≤ 0.1625	≤ 0.1626
HuCCA-1	staurosporine	24	≥ 10	1.507
HuCCA-1	Sorafenib	24	≥ 10	≥ 10
HuCCA-1	PD0325901	24	≥ 10	≥ 10
HuCCA-1	Simvastatin	24	1.651	N.D.
HuCCA-1	Vandetanib	24	≥ 10	0.8091
HuCCA-1	CytochalasinD	24	0.6202	≤ 0.1625
RBE	staurosporine	24	≥ 10	0.8979
RBE	Sorafenib	24	≥ 10	≥ 10
RBE	PD0325901	24	≥ 10	0.5887
RBE	Simvastatin	24	≥ 10	4.548
RBE	Vandetanib	24	≥ 10	0.7983
RBE	CytochalasinD	24	1.749	≥ 10
KKK-D068	staurosporine	48	0.6783	0.287
KKK-D068	Sorafenib	48	≥ 10	3.639
KKK-D068	PD0325901	48	2.023	0.3155
KKK-D068	Simvastatin	48	4.927	≥ 10
KKK-D068	Vandetanib	48	≥ 10	≥ 10
KKK-D068	CytochalasinD	48	≤ 0.1625	0.4848
KKU-213	staurosporine	48	0.8755	0.5637
KKU-213	Sorafenib	48	0.5464	N.D.
KKU-213	PD0325901	48	0.264	0.3471
KKU-213	Simvastatin	48	1.873	≥ 10
KKU-213	Vandetanib	48	≥ 10	2.639
KKU-213	CytochalasinD	48	1.163	0.4538
KKK-D131	staurosporine	48	0.8986	0.5333
KKK-D131	Sorafenib	48	0.796	0.6478

KKK-D131	PD0325901	48	1.751	≥ 10
KKK-D131	Simvastatin	48	≥ 10	1.364
KKK-D131	Vandetanib	48	9.887	0.7351
KKK-D131	CytochalasinD	48	5.17	≤ 0.1625
HuCCA-1	staurosporine	48	≥ 10	1.474
HuCCA-1	Sorafenib	48	6.773	9.001
HuCCA-1	PD0325901	48	≥ 10	≥ 10
HuCCA-1	Simvastatin	48	1.684	N.D.
HuCCA-1	Vandetanib	48	≥ 10	0.7747
HuCCA-1	CytochalasinD	48	0.9902	0.9283
RBE	staurosporine	48	≥ 10	1.019
RBE	Sorafenib	48	5.299	≥ 10
RBE	PD0325901	48	1.502	0.6818
RBE	Simvastatin	48	≥ 10	4.855
RBE	Vandetanib	48	9.897	0.7842
RBE	CytochalasinD	48	0.6199	≥ 10
KKK-D068	staurosporine	72	0.1267	0.3878
KKK-D068	Sorafenib	72	0.2768	4.675
KKK-D068	PD0325901	72	0.322	0.3186
KKK-D068	Simvastatin	72	1.261	≥ 10
KKK-D068	Vandetanib	72	≤ 0.1625	≥ 10
KKK-D068	CytochalasinD	72	≥ 10	0.4843
KKU-213	staurosporine	72	0.9049	0.6079
KKU-213	Sorafenib	72	≥ 10	≤ 0.1625
KKU-213	PD0325901	72	≤ 0.1625	0.3227
KKU-213	Simvastatin	72	3.05	4.841
KKU-213	Vandetanib	72	1.833	4.003
KKU-213	CytochalasinD	72	1.3	0.645
KKK-D131	staurosporine	72	≤ 0.1625	0.5541
KKK-D131	Sorafenib	72	9.627	2.464
KKK-D131	PD0325901	72	7.372	≥ 10
KKK-D131	Simvastatin	72	≥ 10	5.301
KKK-D131	Vandetanib	72	≥ 10	1.66
KKK-D131	CytochalasinD	72	0.2616	≤ 0.1625
HuCCA-1	staurosporine	72	≥ 10	1.351
HuCCA-1	Sorafenib	72	6.292	≥ 10
HuCCA-1	PD0325901	72	≤ 0.1625	≥ 10
HuCCA-1	Simvastatin	72	1.653	N.D.
HuCCA-1	Vandetanib	72	≥ 10	0.8006
HuCCA-1	CytochalasinD	72	1.082	1.687
RBE	staurosporine	72	N.D	0.9887
RBE	Sorafenib	72	5.884	≥ 10
RBE	PD0325901	72	2.693	0.7667
RBE	Simvastatin	72	≥ 10	4.795
RBE	Vandetanib	72	5.888	0.843
RBE	CytochalasinD	72	N.D	9.825

* N.D. = cannot be determined due to curve fitting issue
